# Suicidal continuum (ideation, planning, attempting) in an Islamic country; which should be focused on?

**DOI:** 10.5249/jivr.v13i1.1556

**Published:** 2021-01

**Authors:** Mohamad Khajedaluee, Majid Khadem-Rezaiyan, Lida Jarahi, Hoda Khatibi-Moghadam, Afsaneh Faridpak

**Affiliations:** ^*a*^ Department of Community Medicine and Public Health, Faculty of Medicine, Mashhad University of Medical Sciences, Mashhad, Iran.; ^*b*^ Department of Psychiatry, Faculty of Medicine, Mashhad University of Medical Sciences, Mashhad, Iran.; ^*c*^ Vice Chancellor for Health, Bam University of Medical Sciences, Kerman, Iran.

**Keywords:** Suicide, Ideation, Attempt, Plan, Suicidal behavior

## Abstract

**Background:**

The aim of this study was to identify the characteristics of suicidal ideation (SI), suicidal plan (SP), and suicide attempt (SA) in patients who had survived suicide attempts.

**Methods:**

In a one-year cross-sectional design in Khorasan Razavi province, all suicide attempters who were referred to urban and rural health care centers, hospital’s emergency rooms and agreed to participate in the study were included. The previous twelve-month SI, SP and lifelong SA (prior to the current suicide attempt) were obtained.

**Results:**

The mean age of 856 included individuals was 24.2±8.3 years. The majority (652,76.4%) were females. Half of them were first-time suicide attempters. The mean age of first SI was 22±7.7; SP 22±7.9; and SA 22.2±8 years. The twelve-month prevalence of SI and SP prior to the current suicide attempt was 30% and 26.7%, respectively. Males, unlettered, wedded, and employees were significantly older at their first time SI, SP, and SA (all p less than 0.001). SI (25,44.6%), SP(25,47.2%) and SA(34,75.6%) were more prevalent in widow/divorced individuals(all p-values less than 0.02). SI (OR=53.4,CI95%=33.6-85) increased the risk of SP, and SP(OR=6.7,CI95%=4.5-9.9) increased the risk of SA.

**Conclusions:**

SI seems to be a more important predictor of suicide compared to SP, however, the fact that a significant number of attempters had not any previous detectable suicidal ideation or plan, indi-cates particular clinical considerations. We need to have some presuppositions about the factors leading to unplanned and unthoughtful suicide attempts.

## Introduction

Suicide is a global public health issue. ^1^ Suicide rates have increased by 60% worldwide in the last 45 years.^2^ World Health Organization (WHO) has estimated that more than 1.5 million individuals will die due to attempting suicide in 2020 which equals 2% of all deaths due to various diseases and injuries.^3^ However, there is an iceberg phenomenon: suicide at-tempts are up to 20 times more frequent than complet-ed suicides. 

Many factors affect suicide including psychological, social, biological, cultural and environmental factors.^4^ However, some of them like personality characteristics, psychopathology, parenting style, family function, and substance abuse have got more attention.^5,6^ It should be noted that suicide is not even an isolated event, but a continuum of processes starting from suicidal ideation (SI), suicidal plan (SP), suicide attempt (SA), to suicide completion.^7^ SI which is defined as thoughts of self-killing is an important factor not only for tremendous personal distress, psychological burden or mental-health problems but also for the SP, SA and completed suicide. It has been reported that about one-third of those with SI will finally attempt suicide.^8^ So, it seems that there must be predisposing factors which push forward a person in suicide continuum: not all individuals with SI perform SA. On the other hand, not all individuals with SA had a prior SP or SI. So there may be different risk factors for each of them. To the best of our knowledge, this issue has not been well studied.

There is no doubt that suicide has enduring financial, emotional, and social impact on the friends and family of attempters. Therefore, the prevention of suicide is critical. Some studies have reported that a wide range of interventions including healthy decision making, development of life skills and self-esteem enhancement can reduce suicidal behavior.^9,10^ However, it seems that effective suicide prevention measures will not be effective unless there is a clear understanding of the patterns of SI/SP.^11,12^

Countries of the Eastern Mediterranean Region of the WHO including Iran, are traditionally considered to be characterized by low rates of suicidal behaviors (0-3.1 per 100,000). This could be potentially attributed to the cultural messages and Islamic religious beliefs against suicide.^13^ However, these official reports can be prone to a considerable underestimation due to the stigma of suicide in public culture. A population-based study from the capital of Iran has reported that 12.7% of participants had a lifelong history of SI, 6.2% had a lifelong history of SP, and 3.3% had a lifelong history of SA.^14^ We did not find any study which focuses on suicidal behavior in suicide attempters. So, the main aim of this study was to identify the main epidemiological characteristics of SI, SP, and SA in suicide attempters. Besides we tried to study the whole suicidal continuum (i.e. from SI to SA) to see in what proportion of suicide attempts the whole cycle happens. These can help us to have a better picture of suicidal behavior. 

## Methods 

This study was performed with a cross-sectional design in Khorasan Razavi province from 1 August 2014 to 31 July 2015. Khorasan Razavi is the second-most populous province of the Islamic Republic of Iran (i.e. 8% of the population which equals 6 million individuals) located in the northeast of the country.^15^

All suicide attempters who were referred to urban and rural health care centers and hospital emergency rooms and agreed to participate in the study were included. We used a checklist to evaluate the prevalence of twelve-month SI, SP, and lifelong SA not considering the current admission. We also asked about the age at first SI, SP, and SA. Demographic data including gender, age, marital status, occupational status, and educational level was also obtained.

A trained interviewer filled this questionnaire. The participation in the study was completely free, and oral informed consent (to reduce the dropout rate originated from the social stigma of accepting by signing that he/she had committed suicide) was obtained before filling the questionnaire. Ethical Committee of Mashhad University of Medical Sciences approved the study.

Descriptive analysis (frequency, percentage, mean, standard deviation) and inferential analysis (Mann-Whitney, Kruskal-Wallis, Chi-square, Binary logistic regression) were performed using SPSS 11.5. All tests were two-tailed and p-value<0.05 was considered as statistically significant. 

## Results

Overall, 856 suicide attempters were included in the study. The mean age was 24.2±8.3 years. The majority (652,76.4%) were females. Sixty percent of suicide attempters were married (524) followed by single (272,31.9%) and widow/divorced (57,6.7%) individuals. More than one-third (304,36%) of suicide attempters had a diploma degree, 14.4%(121) higher than a diploma, 26.9%(226) high school and others were illiterate/primary education. Being housewife (375,45.2%) was the most common occupational status followed by students (155,18.7%).

The mean age of thinking about suicide for the first time was 22±7.7 years. This was 22±7.9 years for planning suicide. The study group had performed their first suicide attempt in 22.2±8 years. Thirty percent (250) of the study population had SI in the last 12 months while 26.7% (216) had SP in the last 12 month prior to the current suicide attempt. Half of the study population (342) did not have prior SA. On the other hand, 47.2% (322) had attempted suicide between 1-5 times. The remaining 2.8% (19) had attempted suicide more than six times. No statistical gender difference was found for a previous suicide attempt.

Males were significantly older at their first time “Ideation,” “planning” and “attempting” suicide. Similarly, unlettered, wedded, and employees had the first time SI, SP, and SA at a significantly older age compared to others. Individuals with more than six times of suicide attempt were significantly younger at their first time SI, SP, and SA (Table 1).

**Table 1:** Age in the first suicide ideation, planning and attempting.

		Suicide Ideation	Suicide Plan-ning	Suicide Attempting
**Gender**	**Male**	24.9±9*	25.2±9.5	25.6±9.5
	**Female**	21.2±7.1	21.1±7.2	21.3±7.2
	**p-Value**	<0.001	<0.001	<0.001
**Marital status**	**Single**	18.8±5	18.8±5.1	19.1±5.2
	**Married**	23.6±8a	23.7±8.4^a^	23.8±8.3^a^
	**Widow/Divorced**	23.6±10.3^a^	23±10.3^a^	23.5±10.8^a^
	**p-Value**	<0.001	<0.001	<0.001
**Educational level**	**Cannot read or write**	30.6±12.8	32±13.8	33.2±14.5
	**Primary education**	24.3±9.1^a^	24.6±9.1^a^	24.8±9.3^a^
	**High school**	21.5±6.8^ab^	21.4±6.9^ab^	21.4±6.7^ab^
	**Diploma**	20.5±6.5^ab^	20.5±6.7^ab^	20.5±6.5^ab^
	**Higher than diploma**	22.1±7.2^ab^	22.2±7.2^ab^	22.7±7.5^abd^
	**p-Value**	<0.001	<0.001	<0.001
**Occupational status**	**Jobless**	21.7±8	21.9±8.4	21.8±8
	**Governmental employee**	27.6±12.9^a^	27.3±13.5^a^	27.2±13^a^
	**Manual worker**	23.4±6.5^b^	23.8±8.2	24.5±8.4^a^
	**Self-employed**	26.3±10.1^ac^	25.9±10.2^a^	26±10.4^a^
	**Student**	16.7±3.2^abcd^	16.8±3^abcd^	17±3.3^abcd^
	**Housewife**	22.6±6.7^bde^	22.8±6.9^bde^	23±6.9^bde^
	**p-Value**	<0.001	<0.001	<0.001
**Lifelong number of previous suicide attempts**	**0**	22.5±7	22.7±7	23±7.2
	**1-5**	21.4±8.1	21.2±8.2^a^	21.3±8^a^
	**≥6**	18.2±4.3^a^	17±4.1^ab^	16±3.7^ab^
	**p-Value**	0.02	0.001	<0.01

*Data is represented as Mean±SD; Letters show a statistically significant difference (p<0.05) compared to a)first row, b)second row, c)third row, d)fourth row and e)fifth row of each variable.

No relationship was found between gender and SI, SP, and SA. Most SI was observed in individuals with higher than diploma degree (41,33.9%) while SP (61,28.8%) and SA (96,53.3%) were most prevalent in individuals with a high school degree. However, no statistical difference was found. On the other hand, there was a significant relationship between marital status and all suicidal behavior: SI (25,44.6%), SP (25,47.2%) and SA (34,75.6%) were most prevalent in widow/divorced individuals (Table 2).

**Table 2:** The relationship of main demographic factors with previous suicide ideation (12-month), planning (12-month), and attempt (life-long).

		Suicide Ideation		Suicide Planning		Suicide Attempting	
		Yes	No	Yes	No	Yes	No
**Gender**	Male	55(28.1)*	141(71.9)	52(26.7)	143(73.3)	80(50.6)	78(49.4)
	Female	194(30.1)	450(69.9)	163(26.7)	447(73.3)	260(49.8)	262(50.2)
	**p-Value**	0.59**		**0.99**		**0.92**	
**Marital sta-tus**	Single	84(31)	187(69)	66(25.3)	195(74.7)	111(50)	111(50)
	Married	140(27.3)	373(72.7)	124(25.3)	367(74.7)	195(47.1)	219(52.9)
	Widow/Divorced	25(44.6)	31(55.4)	25(47.2)	28(52.8)	34(75.6)	11(24.4)
	**p-Value**	**0.02**		**0.002**		**0.001**	
**Educational level**	Cannot read or write	6(20)	24(80)	5(17.2)	24(82.8)	8(34.8)	15(65.2)
	Primary education	38(24.1)	120(75.9)	34(22.5)	117(77.5)	54(45.8)	64(54.2)
	High school	67(30.7)	151(69.3)	61(28.8)	151(71.2)	96(53.6)	83(46.4)
	Diploma	92(30.6)	209(69.4)	78(27.1)	210(72.9)	131(50.6)	128(49.4)
	Higher than diploma	41(33.9)	80(66.1)	33(28.2)	84(71.8)	45(47.4)	50(52.6)
	**p-Value**	**0.28**		**0.51**		**0.38**	
**Occupational status**	Jobless	32(34.8)	60(65.2)	28(31.8)	60(68.2)	47(62.7)	28(37.3)
	Governmental employee	8(32)	17(68)	7(28)	18(72)	10(52.6)	9(47.4)
	Manual worker	16(22.2)	56(77.8)	15(21.1)	56(78.9)	33(54.1)	28(45.9)
	Self-employed	37(34.6)	70(65.4)	27(25.5)	79(74.5)	46(54.8)	38(45.2)
	Student	52(33.8)	102(66.2)	44(29.5)	105(70.5)	64(49.6)	65(50.4)
	Housewife	96(26.1)	272(73.9)	87(25.2)	258(74.8)	133(44.6)	165(55.4)
	**p-Value**	**0.16**		**0.63**		**0.91**	

* Data is represented as Frequency (Percentage) ** Chi-square test

**Figure 1-A:**
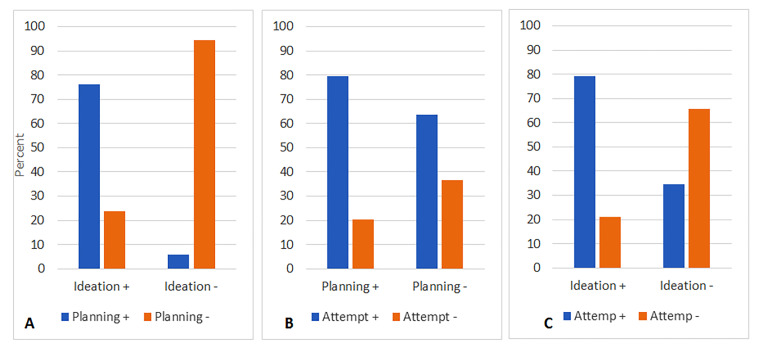
The relationship of suicide ideation and planning; B: the relationship of suicide planning and attempting; C: the relationship of suicide ideation and attempts.

**Figure 2:**
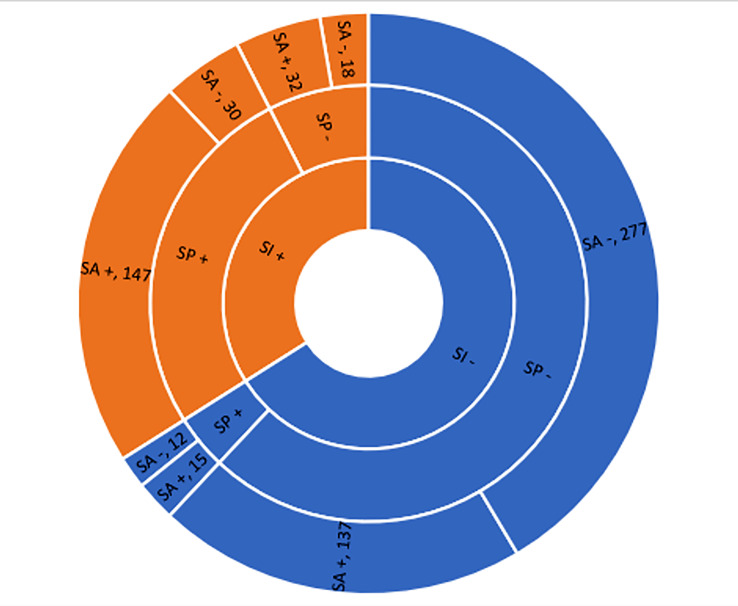
The frequency of individuals in the complete hierarchy of suicide continuum. (SI= Suicide Ideation; SP= Suicide Planning; SA= Suicide At-tempt; Plus sign: having; Minus sign: not having).

Seventy-six percent of individuals with positive SI had a plan to commit suicide (OR=53.4, CI95%=33.6-85, p<0.001). Similarly, 80% of individuals with a positive history of SP had a previous suicide attempt (OR=6.7, CI95%=4.5-9.9, p<0.001). Also, 79% of individuals with positive SI had a previous suicide attempt (OR=7.1, CI95%=4.9-10.3, p<0.001) ( Figure 1 and 2). We performed a deeper analysis, and it was revealed that one-third (137,30%) of individuals with a negative history for both SI and SP had at least one SA. Although 79% (185) of individuals with positive SI finally had SA (OR=7.1, CI95%=4.9-10.3, p<0.001), but 34% (154) of individuals with negative SI had a SA, too. When considering both SI and SP in a logistic regression model, both previously mentioned ORs were reduced. The adjusted OR in committing suicide was 3.7 (CI95%=2.2-6, p<0.001) for SI and 2.6 (CI95%=1.5-4.4, P <0.001) for SP. 

## Discussion

The results of this study that was performed on suicide attempters who referred to all health care centers of Khorasan Razavi Province in one year show that the prevalence of SI and SP in participants was 30% and 26.7%, respectively. SI increased the risk of SP, and SP increased the risk of SA, but although SI seems to be a more important predictor of suicide compared to SP, a significant number of attempters did not have any previous detectable ideation or plan for suicide. In Iran, it has been reported that more than 60% of intentional fatal injuries were due to suicide^16^ The World Mental Health (WMH) Surveys conducted by WHO on 2001-2007, in which 108,705 adults from 21 countries were interviewed using the WHO Composite International Diagnostic Interview showed that there are nearly 15 SAs for each suicide death and the interquartile range of this ratio has been estimated to be between 9.1 to 53.7.^17^ Another study has reported this ratio to be much higher, namely 98.^18^

The majority of our study population (i.e. suicide attempters) were female, married, and graduated with a diploma degree. Female sex, younger age, lower education and poorer income, unmarried status, and unemployment are among the main risk factors for suicidal behaviors.^17^ Although men have a probability of completed suicide four times more than women, women attempt suicide 2-3 times as often as men do,^14,18-21^ which is congruent with our results as we studied the individuals with incomplete suicide (i.e. alive ones). Other studies had found a higher prevalence of suicide attempts in singles which can be due to socio-cultural differences.^22,23^ For example, forced marriage, unwanted pregnancy, being financially dependent on one's husband, and being a housewife with no income may bring a sort of social hopelessness. In this context, suicidal behavior may be an automatic reaction, particularly in the transitional condition during the first several years after marriage for women.

In this study, first time SI, SP, and SA were started in the early 30s. The prevalence of twelve-month SI and SP was 30% and 26.7%, respectively, and half of the participants were first-time suicide attempters which are much lower than the estimation of suicidal behavior for the general population. A WHO multisite intervention study on suicidal behaviors (SUPRE-MISS) which was performed on general population from eight sites (Brazil, China, Estonia, India, Iran, South Africa, Sri Lanka, and Viet Nam) found that SI was (2.6–25.4%), SP (1.1–15.6%), and SA (0.4–4.2%).^24^ Another study in general population of China estimated SI and SA to be 3.9% (CI95%:2.5%-6.0%) and 0.8% (CI95%: 0.7%–0.9%), respectively.^25^ Other Asian countries have reported similar results in their general population (SI:1.7%–15.2%; SA:0.4%–4.2%) which are lower than some Western countries (SI:10%–20%; SA:2%–8%).^25^ A more comprehensive estimation was reported by a systematic review which examined suicide between 1997 and 2007 and found that the lifetime prevalence of SI among adults varied from 3.1% to 56.0% and that of SA was between 0.9% and 19.5%.^26^ In a systematic review of the EMRO region, the lifetime prevalence of SA extracted from community-based surveys varied from 0.72% to 4.2%.^27^ In Iran, only one study has focused on a suicidal continuum in the general population which reported SI, SP, and SA in the last past month to be 5.7%, 2.9%, and 1%, respectively.^28^

The results showed the history of suicide is an important risk factor for suicide after adjustment for sex, age. This correlation was shown in different degrees in several studies like a national study from China: previous suicide attempt, a blood relative with previous suicidal behavior, and a friend with a history of previous suicidal behavior were among the eight main predictors of suicide.^29^ However, it has not reported the effects of SI or SP. Another study found that age below 50 years could be assumed as a predictor of unplanned suicide among suicide ideators.^17^ A more holistic view can be that increasing age is related to more suicide attempts.^19^ This can be explained by the fact that suicide attempters had risk factors but did not have a medical approach, so by passing time the risk factors were consolidated and accumulatively led to a suicide attempt.

The lifetime prevalence of SI and SA in women was higher than the prevalence in men.^25,30,31,32^ Twelve-month SI is also significantly more prevalent among women than men in some countries (2.4% vs. 1.6%).^17^ Although we did not reach a significance level, our findings clinically support previous reports. SP are significantly more common among women than men in developing (0.8% vs. 0.6%), but not in developed countries.

In the Iranian population, the ratios of SA:SP for a lifelong and one-month period is reported to be 1:1.8 and 1:2.9, respectively. The lifelong SP:SI ratio was also reported as 1:2.0.^26^ We found similar results: SP:SI was 1:1.1, SA:SP was 1:1.9 and SA:SI was 1:1.7. WHO estimates that roughly one-third of 12-month individuals with SI develop an SP.^17^ In our study, 76% of suicide ideators developed SP. WHO also estimates that a one-fifth ratio of 12-month ideators makes an SA,^17^ In our study, 79% of suicide ideators developed SA. However, it seems that the critical point can be attributed to individuals with no ideation or planning for suicide. It is reported that unplanned SA constitutes near to 30-40% of all attempts in different countries.^17^ In our study, 35% of individuals with no SI, and 64% of individuals with no SP finally performed an SA. This can show the high impact of SI versus SP in suicidal behavior. It seems that prevention programs should mostly focus on forming SI.

This study is not without limitations. It seems that females are somehow overrepresented in the sample. We tried to cover this by using male interviewers and the assurance of confidentiality. However, free participation is always an ethical code. However, this issue should be considered in the interpretation and generalizability of findings. Besides, it should be remembered that the presence of SI required that a person has “seriously” thought about committing suicide rather than having merely “thoughts of death.” Our analysis was based on retrospective self-reports which could be prone to an underestimation. However, to the best of our knowledge, no other study had focused on suicidal behavior in first-time suicide attempters or patients with the history of several attempts. Since there was not any control group, no comparison could be made with the normal population. However, we believe that this evaluation of non-fatal suicidal behaviors which are the preceding factors and could be the predictors of completed suicide would deserve more investigation.

In conclusion, both SI and SP are the predictors of suicide. The former seems to be of great importance. The fact that a significant number of attempters had not any previous detectable suicidal ideation or plan, indicates some particular clinical considerations. We need to have some presuppositions about the factors leading to unplanned and unthoughtful suicide attempts; factors such as substance abuse, impulse control disorder, and acute life stressors. Being considerate about such factors, and the underlying psychological vulnerabilities provide the chance for managing such conditions more seriously and lower the risk of suicide.

**Acknowledgements**

We appreciate the support of Vice Chancellor for Research of Mashhad University of Medical Sciences. The support of Vice Chancellor for Health and Clinical Research Development Unit of Akbar Hospital (affiliated to Mashhad University of Medical Sciences) is also highly appreciated.
